# CORRIGENDUM: In silico development and validation of Bayesian methods for optimizing deep brain stimulation parameters (2023 *J. Neural Eng.* 20 036015)

**DOI:** 10.1088/1741-2552/aeb32d

**Published:** 2026-10-29

**Authors:** Sumedh S Nagrale, Ali Yousefi, Theoden I Netoff, Alik S Widge

**Affiliations:** 1 Department of Biomedical Engineering, University of Minnesota, Minneapolis, MN, United States of America; 2 Department of Computer Science, Worcester Polytechnic Institute, Worcester, MA, United States of America; 3 Department of Psychiatry and Behavioral Sciences, University of Minnesota, Minneapolis, MN, United States of America

## 1. Statement of correction

An updated multi-stage tie-breaking procedure was applied to correct a technical oversight in our ensemble algorithm code. Figure 6, section 3.10, and supplementary tables S7–S9 are amended below to reflect these recalculated values. All primary scientific conclusions, algorithm rankings, and the superiority of UCB1 remain fully supported and unchanged.

**Figure 6.**
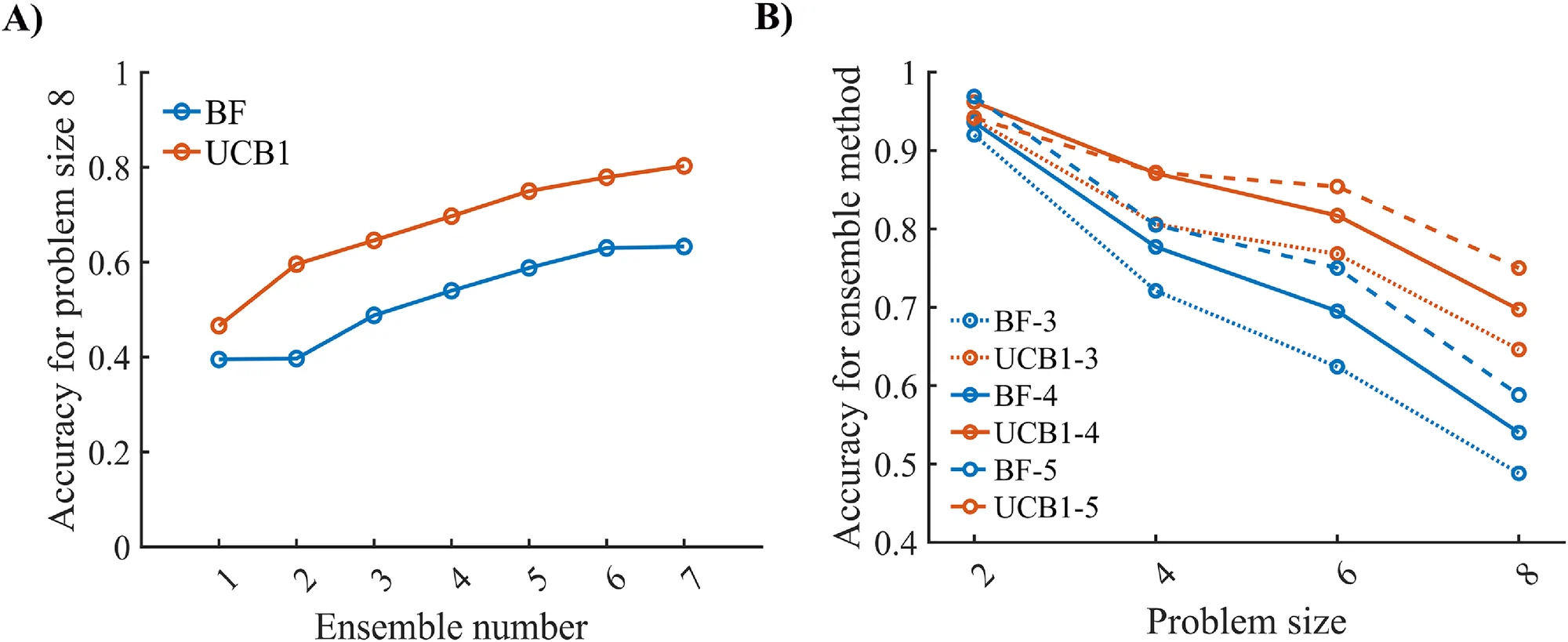
Ensemble algorithm performance. Both UCB1 and BF improve dramatically when optimization is repeatedly run on the same simulated subject. However, UCB1 benefits more, attaining ∼80% accuracy even in the most difficult problems. (A) Accuracy increases with the number of ensembles, showing consistent performance gains throughout the 7 day period, with the steepest improvements occurring within the first three repetitions. (B) Accuracy as a function of problem size. UCB1’s performance advantage over the BF baseline scales with problem complexity; as the search space expands, the performance gap widens. This advantage is maintained regardless of ensembles. Different numbers (and corresponding linestyles) represent the number of ensemble runs, e.g. UCB1-4 represents 4 repeated UCB1 runs on 4 separate days.

## 2. Main paper revisions

### 3.10. Ensemble method for increased accuracy

Ensemble optimization further improved performance (figure 6). In the most difficult problem of eight stimulation sites, UCB1 reached an accuracy of 80% by Day 7, compared to 48% in single-trial simulations. In simpler problems, e.g. four stimulation sites as was originally tested in [37], UCB1’s accuracy exceeded 90%. While performance improved as the ensemble process lengthened from 3 to 7 d, the gains became asymptotic over time. Across the entire ensemble process, UCB1 was the most consistent performer, securing the highest number of ‘wins’ in the majority of problem sizes (table S7). It was also possible to improve performance above 90%, even on difficult problems, by accepting a ‘good enough’ local minimum (e.g. the 2nd or 3rd best rather than optimal stimulation site). This improvement in accuracy was observed across algorithms; Thompson Sampling (TS) in particular showed significant gains when the criteria were expanded beyond the single global optimum. As the ensemble process lengthened, competing algorithms moved closer to the performance of UCB1; however, UCB1 maintained a consistent lead (tables S7–S9) in more complex problem sizes, proving more resilient to the increased search space. In these higher-complexity problems, all adaptive algorithms outperformed the BF baseline (tables S8 and S9).

## 3. Supplementary material revisions

Supplementary Results: Ensemble method, tables for all algorithms

**Table S7.** Ensemble algorithm performance. All algorithms accuracy improves dramatically when optimization is repeatedly run on the same simulated subject. The optimization was repeated 3, 4, and 5 times on the same subject for 1000 iterations. However, for each of the 1000 iterations, the subject (simulated) was selected randomly. UCB1 and Bayes-UCB algorithms performed better for difficult problems. Italic-bold denotes the best performance for a given combination of number of runs and problem size; bold denotes performance exceeding brute force.

	Days				
	Algorithms	3	4	5	Wins
Problem size 2	BF	0.92	0.936	**0.969**	0

	Greedy	0.813	0.825	0.863	0
	ϵ-greedy	0.854	0.846	0.905	0
	UCB1	***0.94***	**0.958**	0.942	1
	Bayes-UCB	0.917	***0.966***	***0.97***	2
	TS-Bernoulli	0.88	0.916	0.925	0
	TS-Poisson	0.878	0.887	0.895	0
	TS-Normal	**0.931**	**0.955**	0.952	0
	C-TS	0.848	0.849	0.893	0

Problem size 4	BF	0.721	0.777	0.805	0

	Greedy	0.649	0.711	0.751	0
	ϵ-greedy	0.675	**0.786**	**0.801**	0
	UCB1	**0.805**	***0.869***	***0.87***	2
	Bayes-UCB	***0.808***	**0.84**	**0.865**	1
	TS-Bernoulli	**0.772**	**0.791**	**0.831**	0
	TS-Poisson	**0.765**	**0.784**	**0.82**	0
	TS-Normal	**0.744**	**0.813**	**0.838**	0
	C-TS	0.692	0.736	0.772	0

Problem size 6	BF	0.624	0.695	0.75	0

	Greedy	**0.671**	**0.726**	0.735	0
	ϵ-greedy	**0.675**	**0.74**	**0.78**	0
	UCB1	***0.76***	***0.814***	***0.855***	3
	Bayes-UCB	**0.756**	**0.79**	**0.812**	0
	TS-Bernoulli	**0.708**	**0.707**	**0.775**	0
	TS-Poisson	**0.714**	**0.762**	**0.788**	0
	TS-Normal	**0.686**	**0.771**	**0.787**	0
	C-TS	**0.649**	0.681	0.746	0

Problem size 8	BF	0.488	0.54	0.588	0

	Greedy	**0.569**	**0.612**	**0.678**	0
	ϵ-greedy	**0.559**	**0.622**	**0.693**	0
	UCB1	***0.636***	***0.696***	***0.744***	3
	Bayes-UCB	**0.563**	**0.604**	**0.637**	0
	TS-Bernoulli	**0.553**	**0.564**	**0.595**	0
	TS-Poisson	**0.574**	**0.606**	**0.672**	0
	TS-Normal	**0.493**	**0.567**	**0.6**	0
	C-TS	**0.489**	**0.563**	0.565	0

Top 2:

Supplementary Results: Ensemble methods, ability to identify near-optimal results

In theory, it may not be necessary to identify the global optimum. In cases where two or more contacts cause cognitive improvement, it may be clinically sufficient to identify one of these, thus providing clinical improvement even if it is not the best possible improvement that an individual patient can get. To that end, we present the various algorithms’ ability to identify one of the 2 or 3 best sites, rather than the global optimum:

**Table S8.** Ensemble algorithm performance for converging to top two optimal stimulation sites. UCB1 shows the highest overall performance across problem sizes. Thompson sampling variants show improvement in accuracy when compared to finding simply the optimal stimulation site, suggesting that TS is converging to near-optimal regions or local minima.

Top 2 optimal stim sites	Days				
	Algorithms	3	4	5	Wins
Problem size 4	BF	0.841	0.879	0.9	0

	Greedy	0.799	0.837	0.863	0
	ϵ-greedy	0.838	**0.897**	0.893	0
	UCB1	**0.891**	***0.947***	***0.935***	2
	Bayes-UCB	***0.904***	**0.91**	**0.929**	1
	TS-Bernoulli	**0.882**	**0.899**	**0.917**	0
	TS-Poisson	**0.865**	**0.883**	**0.908**	0
	TS-Normal	**0.867**	**0.894**	**0.923**	0
	C-TS	0.822	0.865	0.879	0

Problem size 6	BF	0.834	0.872	0.915	0

	Greedy	**0.866**	**0.891**	**0.915**	0
	ϵ-greedy	**0.875**	**0.904**	**0.922**	0
	UCB1	***0.932***	***0.947***	***0.965***	3
	Bayes-UCB	**0.903**	**0.924**	**0.939**	0
	TS-Bernoulli	**0.891**	**0.878**	**0.923**	0
	TS-Poisson	**0.884**	**0.909**	**0.938**	0
	TS-Normal	**0.876**	**0.916**	**0.918**	0
	C-TS	0.821	0.846	0.894	0
Problem size 8	BF	0.625	0.673	0.73	0

	Greedy	**0.742**	**0.748**	**0.812**	0
	ϵ-greedy	**0.717**	**0.756**	**0.823**	0
	UCB1	***0.777***	***0.821***	***0.857***	3
	Bayes-UCB	**0.717**	**0.723**	**0.798**	0
	TS-Bernoulli	**0.684**	**0.692**	0.716	0
	TS-Poisson	**0.722**	**0.756**	**0.793**	0
	TS-Normal	**0.668**	**0.715**	0.729	0
	C-TS	**0.635**	**0.698**	0.686	0

Top 3:

**Table S9.** Ensemble algorithm performance for converging to top three optimal stimulation sites. UCB1 continues to outperform other algorithms across all conditions. Accuracy remains high across most models, with UCB1 consistently achieving over 90% accuracy for a problem size of 8 as the number of days increases.

	Days				
	Algorithms	3	4	5	Wins
Problem size 6	BF	0.898	0.933	0.953	0

	Greedy	**0.92**	**0.943**	**0.968**	0
	ϵ-greedy	**0.94**	**0.959**	**0.97**	0
	UCB1	***0.963***	***0.979***	***0.983***	3
	Bayes-UCB	**0.951**	**0.96**	**0.973**	0
	TS-Bernoulli	**0.935**	0.931	**0.964**	0
	TS-Poisson	**0.936**	**0.946**	**0.965**	0
	TS-Normal	**0.927**	**0.951**	**0.96**	0
	C-TS	0.888	0.907	0.947	0

Problem size 8	BF	0.727	0.763	0.814	0

	Greedy	**0.831**	**0.84**	**0.885**	0
	ϵ-greedy	**0.808**	**0.829**	**0.884**	0
	UCB1	***0.85***	***0.882***	***0.911***	3
	Bayes-UCB	**0.815**	**0.797**	**0.86**	0
	TS-Bernoulli	**0.784**	**0.785**	0.811	0
	TS-Poisson	**0.796**	**0.841**	**0.871**	0
	TS-Normal	**0.758**	**0.798**	**0.818**	0
	C-TS	**0.736**	**0.776**	**0.778**	0

## Data Availability

All data that support the findings of this study are included within the article (and any supplementary files).

